# Prevalence of postural hypotension in primary, community and institutional care: a systematic review and meta-analysis

**DOI:** 10.1186/s12875-020-01313-8

**Published:** 2021-01-02

**Authors:** Sinead T. J. McDonagh, Natasha Mejzner, Christopher E. Clark

**Affiliations:** Primary Care Research Group, University of Exeter Medical School, College of Medicine and Health, St Luke’s Campus, Magdalen Road, Exeter, Devon EX1 2LU England

**Keywords:** Postural hypotension, Orthostatic hypotension, Prevalence, Primary care, Nursing homes, Community

## Abstract

**Background:**

Postural hypotension (PH), the reduction in blood pressure when rising from sitting or lying 0to standing, is a risk factor for falls, cognitive decline and mortality. However, it is not often tested for in primary care. PH prevalence varies according to definition, population, care setting and measurement method. The aim of this study was to determine the prevalence of PH across different care settings and disease subgroups.

**Methods:**

Systematic review, meta-analyses and meta-regression. We searched Medline and Embase to October 2019 for studies based in primary, community or institutional care settings reporting PH prevalence. Data and study level demographics were extracted independently by two reviewers. Pooled estimates for mean PH prevalence were compared between care settings and disease subgroups using random effects meta-analyses. Predictors of PH were explored using meta-regression. Quality assessment was undertaken using an adapted Newcastle-Ottawa Scale.

**Results:**

One thousand eight hundred sixteen studies were identified; 61 contributed to analyses. Pooled prevalences for PH using the consensus definition were 17% (95% CI, 14–20%; I^2^ = 99%) for 34 community cohorts, 19% (15–25%; I^2^ = 98%) for 23 primary care cohorts and 31% (15–50%; I^2^ = 0%) for 3 residential care or nursing homes cohorts (*P* = 0.16 between groups). By condition, prevalences were 20% (16–23%; I^2^ = 98%) with hypertension (20 cohorts), 21% (16–26%; I^2^ = 92%) with diabetes (4 cohorts), 25% (18–33%; I^2^ = 88%) with Parkinson’s disease (7 cohorts) and 29% (25–33%, I^2^ = 0%) with dementia (3 cohorts), compared to 14% (12–17%, I^2^ = 99%) without these conditions (*P* < 0.01 between groups). Multivariable meta-regression modelling identified increasing age and diabetes as predictors of PH (*P* < 0.01, *P* = 0.13, respectively; R^2^ = 36%). PH prevalence was not affected by blood pressure measurement device (*P* = 0.65) or sitting or supine resting position (*P* = 0.24), however, when the definition of PH did not fulfil the consensus description, but fell within its parameters, prevalence was underestimated (*P =* 0.01) irrespective of study quality (*P =* 0.04).

**Conclusions:**

PH prevalence in populations relevant to primary care is substantial and the definition of PH used is important. Our findings emphasise the importance of considering checking for PH, particularly in vulnerable populations, to enable interventions to manage it. These data should contribute to future guidelines relevant to the detection and treatment of PH.

PROSPERO:CRD42017075423.

## Background

Postural, or orthostatic, hypotension (PH), is the fall in blood pressure (BP) when rising from seated or supine to standing [1]. It is associated with an increased risk of falls, cognitive decline, reduced quality of life and mortality [2–5].

Current National Institute for Health and Care Excellence (NICE) hypertension guidelines advise testing for PH in the presence of type 2 diabetes, postural symptoms or aged 80 or over [6]; European guidelines also suggest checking in older people and those with diabetes [7]. Whilst PH is routinely tested for in primary care when symptoms are reported, we have found that it is only considered one third of the time for older people and rarely with diabetes, in the absence of symptoms [8]. Since the majority of people with PH are asymptomatic, they are likely to go undetected under current practices, placing them at avoidable risk of sequelae [5, 9].

In 2011, a consensus definition for PH: a sustained reduction in systolic BP ≥20 mmHg or diastolic BP ≥10 mmHg within 3 min of rising to a standing position, was proposed [1]. However, many other definitions of PH exist; reported prevalence estimates are likely dependent on the definition used, making this a source of variance and uncertainty around diagnosis of PH. Prevalence may also vary depending on the method of BP measurement, population and care setting under investigation. The prevalence of PH has been reported as ranging from 2 to 57% in community settings, primary care and institutional care cohorts [4, 10, 11]; increasing prevalences have been associated with older age, diabetes and hypertension [9, 12–14].

The large variation of reported prevalences may create uncertainty for clinicians as to who should be assessed for PH [15]. By describing the prevalences of PH in settings and conditions relevant to primary care, and identifying factors associated with greater prevalences, we aim to raise awareness of those patients most likely to have asymptomatic PH. Such evidence could counteract clinical inertia and facilitate rational choices, in the face of rising workload, as to when to invest time in testing for PH [16, 17]. Increased recognition of PH would permit appropriate interventions, such as review of medications, to reduce risks of falls and other sequelae [18]. We undertook the following systematic review, meta-analyses and meta-regression to address these questions.

## Methods

### Literature searches

A systematic review was undertaken to determine the prevalence of postural hypotension across care settings. This study was prospectively registered with PROSPERO: CRD42017075423. We searched Medline (including Medline in Process and Old Medline) and Embase from their respective commencement dates until 1st October 2019, using a broad search strategy based on key search terms (Appendix 1). Further studies were identified from the authors’ archives and from reference lists of included studies and review articles. Study titles and abstracts were screened independently by two authors. Disagreements were discussed to reach consensus, with provision for adjudication by a third author, if needed. Two authors assessed and agreed full texts for inclusion, undertook data extraction and assessed study quality; the review process was managed using *Covidence* (Veritas Health Innovation, Melbourne, Australia).

### Inclusion and exclusion criteria

Studies were eligible for inclusion if BP was measured in a lying or seated position followed by standing and using either a manual or automated sphygmomanometer. Eligible study settings were primary care, community or residential/nursing home populations. We identified 78 distinct definitions of PH in scoping studies for this review. To minimise heterogeneity of findings due to definitions, we restricted inclusion to studies which either reported using the consensus definition or adopted a definition encompassed within the consensus definition [1]. Exclusion criteria are summarised in Table 1.

**Table 1:** Exclusion criteria for review

• Definition of PH incompatible with consensus definition • Studies where PH was provoked by tilt table testing or pain stimulation • Studies using continuous or ambulatory BP monitoring for diagnosis of PH • Drug trials • Specific but specialised cohorts, e.g. spinal injuries, multiple sclerosis or HIV • Studies from secondary and tertiary care settings	

### Data extraction

Study level demographics were extracted for care setting, mean age, BP measurement device, resting position (seated or supine) and medical history of hypertension, diabetes, Parkinson’s disease or dementia. Where a range of health status existed within a study population, if more than 50% of the total cohort included individuals with a particular condition, hypertension, for example, we applied the appropriate disease classification, i.e. the cohort would be classed as a hypertensive cohort. Populations were included within the community category, unless specifically selected from a primary care or institutional care setting. The Newcastle-Ottawa Scale (NOS), with questions adapted to PH specific context, was used to assess study quality (Appendix 2). Where multiple reports for a cohort were retrieved, extraction was primarily taken from the main publication, with addition of detail from subsidiary reports where needed.

### Statistical analysis

Pooled estimates of mean prevalences for PH were calculated and compared between settings and populations using meta-analysis of proportions, undertaken in *Stata v16* (Statacorp, Texas, USA) [19]. Random effects models were used throughout due to anticipated heterogeneity between included studies. Statistical heterogeneity was assessed using the I^2^ statistic, and explored with sensitivity analyses, using meta-analysis, based on care setting, disease status or BP measurement method. We also conducted sensitivity analyses according to the definition of PH, i.e. whether PH was reported using the consensus definition or a definition that fell within the consensus definition parameters but did not fully meet them (e.g. by measuring BP over less than three minutes of standing). Univariable meta-regression analyses were undertaken to examine association between study level factors [mean age, percentage of females, mean absolute resting systolic BP, care setting, BP measurement method (auscultatory or oscillometric) or position (seated or supine), disease status (hypertension, diabetes, Parkinson’s disease or dementia)] and prevalence of PH [20]. Factors suggesting univariable associations with PH (using *P* < 0.1) were entered into multivariable models, with a priori inclusion of age, care setting and presence of diabetes and hypertension. Publication bias was assessed visually using funnel plots and quantified with the Egger test [21].

## Results

**Fig. 1:**
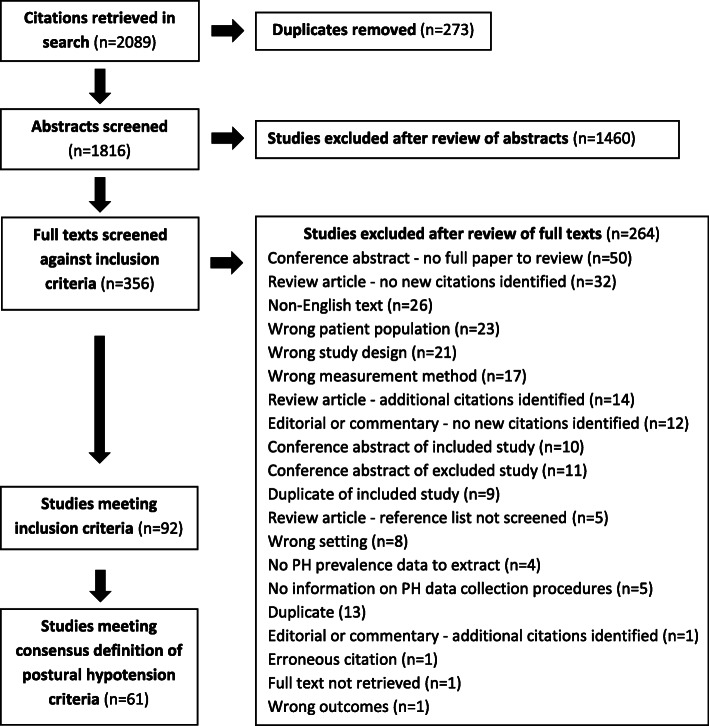
Flow chart illustrating the process of inclusion or exclusion in this prevalence of postural hypotension systematic review

Searches identified 1816 unique citations; 356 full texts were reviewed; 92 studies met inclusion criteria, but only 61 fell within the consensus definition of PH, thus contributing to the meta-analyses. Reasons for the exclusion of studies are summarised in Fig. 1.

### Description of studies

All included studies were cross-sectional or cohort studies, with cohort size ranging from 40 to 32,797 participants (Table 2). On quality assessment, areas of low quality (defined as falling below the median NOS total score of 8; range: 3–10) were notable in categories relating to the response rates of participants and in comparability between respondents and non-respondents (usually due to lack of information provided), and the use of non-validated methods for BP measurement (Table 3.).

**Table 2:** Studies included in meta-analysis for consensus definition of postural hypotension

Study	Subjects	Age (mean or range; years)	BP measurement method	Prevalence of PH (%)
**Institutional care**				
**Reported as consensus definition of PH**				
Hommel 2016 [22]	Male and female nursing home residents in the Netherlands, receiving long term care and using antiparkinsonian medication	78.8	After 10 min of supine rest and at 1 and 3 min after standing up, BP was measured using a routine sphygmomamometer	51.6
**Not reported but fits consensus definition of PH**				
Enrique Asensio 2011 [23]	Male and female Mexican residents of public or private care institutions, aged over 65 years and able to sign informed consent	82.4	After 5 min of seated rest and at 1 and 3 min of standing up, BP was measured using an aneroid oscillometric sphygmomanometer	29.5
Valbusa 2012 [24]	Male and female nursing home residents in France and Italy, aged over 80 years and able to sign informed consent	88	After 10 min of seated rest and at 1 and 3 min of standing up, BP was measured using the Colson DM-H20 automated oscillometric device (Dupont Médical, Frouard, France). All measurements were repeated three times, with intervals of 3 min on the left arm in a sitting position	18.0
**Primary care**				
**Reported as consensus definition of PH**				
Bouhanick 2014 [25]	Male and females living in France with type 2 diabetes, aged over 70 years with relatively preserved autonomy (Activity of Daily Living Score > 3/6)	77.0	After 5 min of supine rest and at 1, 3 and 5 min after standing up, BP was measured. The BP device was not stated	27.0
Fleg 2016 [26]	Male and female participants with type 2 diabetes and a glycohaemoglobin level ≥ 7.5%, aged 40–79 years with cardiovascular disease or aged 55–79 years with anatomic evidence of subclinical atherosclerosis, albuminuria, left ventricular hypertrophy or ≥ 2 additional risk factors for cardiovascular disease, attending 77 sites across the U.S.A and Canada	40.0–79.0	BP was measured three times, at 1 min intervals, after 5 min of seated rest and on standing, using an automated oscillometric device (Omron HEM-907; Omron Healthcare Co. Kyoto, Japan)	17.7
Hirai 2009 [27]	Male and female participants with type 1 or 2 diabetes living in Wisconsin	45.4	BP was measured using a standard mercury sphygmomanometer during supine rest and repeated within 3 min after participants were asked to stand up	16.1
Klanbut 2018 [28]	Male and female participants with Parkinson’s disease (Hoehn and Yahr stage I-IV), stable on drug therapy or not received any drug modifications for 4 weeks prior to enrolment, attending King Chulalongkorn, Thailand	65.5	After 10 min of seated or supine rest, and within 3 min of standing, BP was measured using an automated sphygmomanometer (Omron HEM-7200)	22.0
Kleipool 2019 [29]	Male and female participants (from the Amsterdam Dementia cohort) with subjective cognitive decline, mild cognitive impairment or dementia attending a memory clinic.	63.9	After 5 min of supine rest, and at 1 and 3 min after standing, BP was measured. The BP device was not stated	29.0
Merola 2016 [30]	Male and females with idiopathic Parkinson’s disease (Hoehn and Yahr stage I-IV), aged 30–85 years, on dopaminergic treatment for at least 4 weeks prior to study enrolment, attending two specialised Movement Disorder Centres in the USA and Italy	30.0–85.0	After 10 min of seated and supine rest and at 1 and 3 min after standing, BP was measured in the left arm using an automated sphygmomanometer (Omron, HEM-7200; Omron Healthcare Co. Kyoto, Japan). The average of two BP measurements were used for both seated and supine rest	30.6
Romagnolo 2019 [31]	Male and female participants, with idiopathic Parkinson’s disease, aged between 30 and 85 years old, attending a Movement Disorder Center in Italy. Participants must have been taking stable doses of dopaminergic treatment for at least 4 weeks prior to enrolment in the study	65.06	BP was measured after 10 min supine rest and at 1, 3 and 5 min after standing using a mercury sphygmomanometer	34.
Sonnesyn 2009 [32]	Male and female participants with first time diagnosis of mild dementia (mini mental state score of at least 20) with referrals to outpatient clinics in geriatric medicine, old age psychiatry and neurology and living in Norway	n/a	After supine rest (or in some cases, seated rest) and then once within 3 min of standing, BP was measured using an analogue sphygmomanometer	31.0
Wecht 2016 [33]	Male and female veterans attending an urban Medical Centre, U.S.A	21.0–88.0	After 10 min supine rest and during 10 min of standing, BP was measured in the right arm at 1 min intervals using an automated sphygmomanometer (Dynamap Pro 300; GE Healthcare, Buckinghamshire, UK)	14.0
**Not reported but fits consensus definition of PH**				
Alli 1992 [34]	Male and female participants aged over 65 years, attending general practices in Italy	72.7	BP was measured in the sitting position, after 5 min of supine rest and 30 s after standing up using a mercury sphygmomanometer	5.9
Atli 2006 [35]	Male and female participants aged over 65 years, attending the outpatient clinic of Ankara University School of Medicine, Department of Geriatric Medicine, Turkey	68.0	After 20 min of supine rest and 3 min after standing, BP was measured using a manual sphygmomanometer	14.8
Bengtsson Lindberg 2015 [36]	154 male and female dementia patients (50 with Alzheimer’s disease, 54 with Alzheimer’s disease with vascular components, 50 with dementia with Lewy bodies) attending a Memory Clinic and 50 controls, in Sweden	n/a	After 10 min of supine rest, immediately after standing and at 1, 3, 5 and 10 min of standing, BP was measured using a validated digital sphygmomanometer (Omron M5–1; Omron Healthcare Co. Kyoto, Japan)	34.3
Clara 2007 [10]	Male and female participants aged over 55 years, living in Portugal, attending primary healthcare centres in the community	n/a	After 5 and 7 min of seated rest, and at 2 and 5 min after standing, BP was measured using a calibrated mercury sphygmomanometer	2.4
Hiorth 2019 [37]	Male and female participants, with incident, drug-naïve Parkinson’s disease, residing in Southern and Western Norway	67.8	BP was measured in a supine position and after 1 min of standing, using a manual sphygmomanometer	19.5
Kamaruzzaman 2010 [38]	Females aged 60–80 years of age, living in the U. K and attending general practices	60.0–80.0	Two sitting BP measurements, followed by two standing measurements were recorded at 1 min intervals. The BP device was not stated	28.1
Liepelt Scarfone 2015 [39]	Male and female patients with Parkinson’s disease, aged over 50 years, attending the outpatient clinic of the Department of Neurodegeneration, University of Tübingen, Germany	> 50.0	After 2 min supine rest and after 2 min of standing, BP was measured manually	17.0
Liu 2016 [40]	Male and female participants attending a community health centre in Chengdu, China	64.8	After 5 min of seated rest, BP was measured in the right arm, twice (at 1 min apart) using a calibrated electronic device (Omron HEM-7200; Omron Healthcare Co. Kyoto, Japan). After 10 further min of supine rest, and at 30 s and 2 min after standing, BP was recorded again and these measurements were used to determine postural hypotension	5.6
Masuo 1996 [41]	Individuals living in Japan, aged ≥65 years with normotension (≤ 140/90 mmHg) or established hypertension (160/95 mmHg) treated with calcium channel blockers, beta blockers, alpha blockers, angiotensin converting enzyme inhibitors or diuretics after a 1 month placebo run-in period	≥ 65.0	BP was measured after 5 min of seated rest, 10 min supine rest and after standing for 2 min (in this order). The BP device was not stated	12.0
Oishi 2016 [42]	Male and female patients, aged over 70 years, who visited a hospital in Japan for day care, programmed for those with dementia	84.0	BP was measured using a validated electronic device (Parama-Tech PS-501) in the supine position after a few minutes of rest and immediately on standing and at 1, 3 and 5 min after standing	26.6
Perez Orcero 2016 [43]	Male and female patients, aged over 80 years, able to stand for 5 or more minutes and attending an urban primary health care centre or treated at home by a family doctor or nurse were included	85.2	After 5 min of rest in the supine position, 2 separate BP readings were taken 1 min apart, and then BP was measured immediately on standing and at 1, 3 and 5 min after standing, using a validated and calibrated oscillometric Omron 705-CP device (Omron Healthcare Co. Kyoto, Japan)	30.7
Van Hateren 2012 [44]	Male and female patients, aged ≥70 years, with type 2 diabetes attending general practices in the Netherlands	75.0	After 5 min of rest, two BP measurements were performed in the supine position and at 1 and 3 min following standing, using a validated A&D digital monitor (UA-767 plus 30). The mean of the two measurements at each time point was calculated	24.3
Walczak 1991 [45]	Male and female individuals, aged 63–93 years attending a day centre	63.0–93.0	After 20 min of supine rest and after 2 min of standing, BP was measured using a standard sphygmomanometer	28.4
Zhu 2016 [46]	Multiethnic Asian ambulatory male and female patients, aged ≥65 years, attending a typical public primary care clinic located in the mideastern part of Singapore	74.6	After 5 min of supine rest, BP was measured 3 times in the right arm. In addition, BP was measured at 1 and 3 min following standing using a calibrated DINAMAP BP machine (Procare 100; GE Healthcare, Little Chalfont, Buckinghamshire, UK)	11.0
**Community care**				
**Reported as consensus definition of PH**				
Cremer 2017 [47]	Male and female participants, aged over 65 years, living in three cities in France (Bordeaux, Dijon and Montpellier.)	> 65.0	After 5 min of supine rest and immediately on standing, BP was measured once using an automated oscillometric device (Omron CP750, Omron Healthcare Co. Kyoto, Japan)	13.0
Drozdz 2016 [48]	Male and female participants, aged over 18 years, with New York Heart Association class II-III chronic heart failure, with left ventricular ejection fraction < 40% under stable conditions, with no cardiovascular interventions in the past 3 months and stable on pharmacological treatment in the 4 weeks prior to study enrolment	63.3	After 10 min of supine rest and within 3 min of standing, BP was measured using a validated oscillometric device (Omron M6; Omron Healthcare Co. Kyoto, Japan)	10.0
Foster-Dingley 2018 [49]	Male and female participants, aged at least 75 years, using antihypertensive medication, with a systolic BP 160 mmHg or less and a Mini Mental State examination score of 21–27. Participants were residing in the Netherlands and did not have serious cardiovascular disease or a clinical diagnosis of dementia	81.0	After 5 min of seated rest, BP was measured twice (separated by 1–2 min) and within 3 min of standing, BP was measured 3 times, on the right arm. An automatic electronic sphygmomanometer (Omron M6 comfort; Omron Healthcare, Inc., Lake Forest, Ilinois, USA)	47.4
Hiitola 2009 [50]	Male and female home-dwelling participants, aged over 75 years, living in Kuopio in Eastern Finland	81.0	After 10 min of rest, BP was measured in the supine, seated and standing positions (at 1 and 3 min) by a trained nurse using a calibrated mercury column sphygmomanometer	34.0
Kartheek 2011 [51]	Male and female participants, aged 20–90 years, of mixed socioeconomic status, living in Kurnool and Kadapa district urban areas of India. Participants were non-smokers and free of any cardiorespiratory disease	20.0–90.0	BP was measured in the right arm using a mercury sphygmomanometer after 5 min of supine rest and at 1 and 3 min following standing. The average of two readings were taken to determine BP	8.9
Mendez 2018 [52]	Male and female participants aged over 55 years, residing in Venezuela	66.7	BP was measured in the supine position and at 1 and 3 min after standing using the same oscillometric device (Dinamap 8100, Critikon Inc., Tampa, FL, USA)	19.3
Nguyen 2017 [53]	Male and female participants, aged 60 years or older, able to communicate and sit and stand in 3 min, residing in Ben Tre, a Southern Province Vietnam	70.4	After at least 15 min of rest, two seated BP measurements were obtained, separated by 5 min. The mean of the two sitting BPs were used for analysis. BP measurements were repeated after standing for 3 min. BP was measured using a calibrated Omron electronic sphygmomanometer (model HEM 7130, OMRON Corp, Kyoto, Japan)	14.9
Putnam 2018 [54]	Male and female participants, aged 70 years or older, residing in Tanzania	72–80	BP was measured using a calibrated A&D Medical UA-1020 Digital BP monitor in the supine position, followed by 30 s, 1, 2 and 3 min after standing	26.8
Rockwood 2012 [55]	Elderly male and female participants living in Canada	83.2	BP was measured in supine and standing positions (or seated in those unable to stand) within 3 min using a sphygmomanometer	17.7
Veronese 2014 [56]	Male and female participants, aged ≥65 years, living in Italy	73.8	BP was measured 3 times in the right arm, with 30 s between each measurement, using a mercury sphygmomanometer (Erkameter 300) with subjects in a supine position. BP measurements were also recorded at 1 and 3 min after standing	32.2
Wolters 2016 [57]	Male and female participants, aged ≥55 years, from the Ommoord area, a suburb of Rotterdam, Netherlands	68.5	After 5 min of supine rest, the mean of 2 BP measurements were recorded. BP was also recorded following 1, 2 and 3 min of standing using an automatic machine (Dinamap, Critikon)	12.5
**Not reported but fits consensus definition of PH**				
Assantachai 1998 [58]	Male and female participants, aged over 60 years, living in Bangkok and able to perform postural change from lying to standing by themselves	60.0–96.0	After 10 min of supine rest, BP was measured twice using a digital sphygmomanometer. BP was then recorded twice during 1–2 min of standing,	12.9
Bell 2016 ARIC [59]	Male and female participants, aged 45–64 years, living in 4 U.S.A communities: Forsyth County, Jackson, suburban Minneapolis and Washington County	54.0	After 20 min of supine rest, BP was measured every 30 s for 2 min (2–5 measurements, 90% of participants had ≥4 measurements) using a Dinamap 1846 SX automated oscillometric device. BP was then measured repeatedly for the first 2 min after standing (2–5 measurements, 91% of participants had ≥4 measurements)	7.5
Bell 2016 CHS [59]	Male and female community-dwelling individuals, aged over 65 years, living in 4 U.S.A communities: Pittsburgh, Forsyth County, Sacramento County and Washington County	73.0	After at least 20 min of supine rest and after 3 min of standing, BP was measured using a mercury sphygmomanometer (Baumanometer, W.A. Baum, Copiague, NY)	18.2
Cilia 2015 [60]	Male and female patients with Parkinson’s disease for ≥20 years	n/a	BP protocol or device not reported	16.0
Cohen 2015 [11]	Male and female participants, aged over 65 years, independent in ambulation and attending a primary care clinic in Israel	73.6	BP was measured every min during 10 min of supine rest and within 1 min of standing and repeated at 1 min intervals another 6 times. BP was measured using an automatic Scholar III 507 EL monitor (CSI - Criticare Systems, Inc.)	56.8
Curreri 2016 [61]	Male and female community-dwelling individuals, aged ≥65 years, living in Camposampiero and Rovigo, Italy	71.4	After at least 5 min of supine rest, BP was measured 3 times in the right arm, at 30 s intervals, using a mercury sphygmomanometer (Erkameter 300). BP was then measured after 1 and 3 min of standing	18.3
Ensrud 1992 [62]	Female participants, aged over 65 years, residing in the U.S.A: Portland, Minneapolis, Baltimore and Monongahela Valley, near Pittsburgh	71.7	After 5 min of supine rest and after 1 min of assuming a standing position, BP was measured in the right arm using a Baum mercury sphygmomanometer	20.0
Fan 2010 [63]	Male and female rural community residents, aged 40–75 years, living in Xinyang County, China	40.0–75.0	After 15 min of supine rest, BP was measured 3 times in the right arm at 30 s intervals and at 30 s and 2 min after standing	22.6
Fedorowski 2010 [64]	Middle-aged male and female individuals, living in Sweden	45.7	After 10 min of supine rest and at 1 min after standing up, BP was measured using a mercury sphygmomanometer	6.1
Frewen 2014 [65]	Community-dwelling male and female individuals, aged over 50 years, living in the Republic of Ireland	63	After 30 min of seated rest, 2 BP measurements were recorded, separated by 1 min, using an automatic digital BP monitor (Omron M10-IT; Omron Healthcare Co. Kyoto, Japan). After 1 min of standing, a single BP measurement was also recorded	6.0
Gangavati 2011 [66]	Male and female individuals, aged over 70 years, who were able to understand and communicate in English, walk 20 ft. without assistance and living in Boston, U.S.A	78.1	After 5 min of supine rest, 2 BP measurements were recorded, separated by 1 min, using a standard sphygmomanometer. The mean of the 2 measurements were used for analysis. BP was also recorded at 1 and 3 min after standing	5.8
Lampela 2013 [67]	Male and female participants, aged ≥75 years, living in Kuopio, Finland, including mainly home-dwelling individuals, but part of the sample were living in institutional care	≥ 75.0	BP was measured after 10 min of rest in the supine position, 1 min after sitting and after 1 and 3 min of standing using an electronic sphygmomanometer or mercury where needed (e.g. if atrial fibrillation was present)	32.7
Luukinen 1999 [68]	Male and female home-dwelling participants, aged over 70 years, living in 5 rural municipalities in northern Finland	76.0	After 5 min of supine rest and at 1 and 3 min after standing up, BP was measured in the right arm using a mercury manometer	30.3
Luukkonen 2017 [69]	Male and female home care clients, aged over 75 years, living in Eastern and Central Finland	84.5	After 10 min of supine rest, the first BP was measured. The second BP was measured in the seated position, followed further BP measurements at 1 and 3 min after standing using an automated BP device	35.7
Mader 1987 [70]	Independent, community living individuals, aged over 55 years who utilise the free health screening services of Santa Monica Senior Health and Peer Counseling Center, U.S.A	69.8	After 5 min of supine rest, 3 BP measurements were recorded over a 5 min period using a mercury manometer (Baumanometer). The mean of the second and third BP measurements were used. BP was also measured 1 min after standing	2.0
Masaki 1998 [71]	Males of Japanese ancestry, aged 45–68 years and living on the island of Oahu, Hawaii	71–93	After at least 15 min of supine rest and after 3 min of standing, BP was measured using a standard mercury sphygmomanometer	6.9
O’Connell 2015 [72]	Community-dwelling male and female individuals, aged over 50 years, living in the Republic of Ireland	63.0	After at least 30 min of seated rest, 2 BP measurements were recorded and the mean was used for analysis. A single BP measurement was also recorded after 1 min of standing using an automated BP device (Omron M10-IT; Omron Healthcare Co. Kyoto, Japan)	6.1
Ong 2017 [73]	Male and female individuals, aged over 60 years, living in the community (day care centres, nursing homes and institutions were included) who were citizens or permanent residents in Singapore	> 60.0	Two sitting BP measurements were recorded and the mean was used for analysis. Standing BP was also measured after 2 min using a standard electronic sphygmomanometer (Omron HEM-7211; Omron Healthcare Co. Kyoto, Japan)	7.8
Shin 2004 [74]	Male and female Korean individuals, aged 40–69 years, living in an industrialised community 32 km southwest of Seoul, South Korea (Ansan) and in a rural setting, 100 km south of Seoul (Ansung)	40.0–69.0	After at least 5 min of supine rest, BP was measured 3 times at 30 s intervals and the mean was used for analysis. Standing BP measurements were recorded at 0 and 2 min after standing	13.9
Vanhanen 1996 [75]	Male and female individuals, aged over 60 years, with isolated systolic hypertension (sitting SBP 160–219 mmHg, DBP < 95 mmHg and a standing SBP ≥ 140 mmHg)	70.0	BP was measured twice after 2 min of rest in the supine position, twice after 5 min of rest in the seated position and twice after 2 min in the standing position using conventional sphygmomanometry	15.0
Velilla Zancada 2017 [76]	Male and female individuals, aged over 18 years, living in Cantabria, Spain	48.5	After 5 min of rest, 3 BP measurements in the dominant arm were recorded in the sitting position using the validated and semiautomatic device (Omron 705 CP; Peróxidos Farmacéuticos S.A. Barcelona, Spain) and the mean of the final 2 measurements were used for analysis. BP was also measured at 1 and 3 min after standing	7.4
Viramo 1999 [77]	Male and female, home-dwelling and institutionalised individuals, born in 1920 or earlier and living in 5 rural municipalities around the town of Oulu, Northern Finland	> 70.0	After 5 min of supine rest and at 1 and 3 min after standing, BP was measured in the right arm. The BP device was not stated	28.7
Wu 2009 [78]	Male and female community-dwelling individuals, with normal glucose tolerance, pre-diabetes and diabetes, aged ≥20 years, living in Tainan, a city in southern Taiwan	≥ 20.0	Two seated BP measurements were recorded, with at least 5 min intervals, after at least 15 min rest using a DINAMAP vital sign monitor (model 1846SX; Critikon, Irvine, CA). BP was also measured twice in the supine position followed by measurements at 1 and 3 min after standing	15.9
Yap 2008 [79]	Male and female individuals, aged over 55 years, living in the south-east region of Singapore	65.5	After at least 10 min of rest, BP was measured up to 3 times, at 30 s intervals, in the right arm in the supine, seated and standing positions using a standard mercury sphygmomanometer. The mean of the two closest readings was used for analysis	16.6

*BP* blood pressure, *s* seconds, *min* minutes

**Table 3:** Newcastle-Ottawa Scale quality assessment of included studies

Study	Selection				Comparability	Outcome		Newcastle Ottawa Score Total
	Representativeness of the sample (true representation of target population)	Sample size (justified and satisfactory)	Non-participants (respondents vs. non respondents and response rate)	Ascertainment of the exposure (BP measurement method)	Comparability of groups on basis of design or analysis (study controls for important factors such as BP, age, gender, anti-hypertensive medication use)	Assessment of the outcome (PH as n/N and/or %)	The outcome is presented in full (no unexplained omissions from dataset)	
Alli 1992 [34]	*	*	/	*	*	**	*	7
Assantachai 1998 [58]	*	*	/	*	**	**	*	8
Atli 2006 [35]	*	/	/	*	/	**	*	5
Bell 2016 ARIC [59]	*	*	*	*	**	**	*	9
Bell 2016 CHS [59]	*	*	*	*	**	**	*	9
Bengtsson Lindberg 2015 [36]	*	*	/	*	*	**	*	7
Bouhanick 2014 [25]	*	*	/	*	**	**	*	8
Cilia 2015 [60]	/	*	/	*	*	**	/	5
Clara 2007 [10]	*	*	/	*	/	*	*	5
Cohen 2015 [11]	*	*	/	*	/	**	*	6
Cremer 2017 [47]	*	*	/	*	**	**	*	8
Curreri 2016 [61]	*	*	/	**	**	**	*	9
Drozdz 2016 [48]	/	/	/	**	/	**	*	5
Enrique Asensio 2011 [23]	*	*	/	**	/	**	*	7
Ensrud 1992 [62]	/	*	/	*	**	**	*	7
Fan 2010 [63]	*	*	*	*	**	**	*	9
Fedorowski 2010 [64]	*	*	*	/	**	**	*	8
Fleg 2016 [26]	/	*	*	*	**	**	*	8
Foster-Dingley 2018 [49]	*	*	/	*	**	**	*	8
Frewen 2014 [65]	*	*	/	/	**	**	*	7
Gangavati 2011 [66]	*	*	/	**	*	**	*	8
Hiitola 2009 [50]	*	*	*	**	**	**	*	10
Hiorth 2019 [37]	*	*	*	*	*	**	*	8
Hirai 2009 [27]	*	*	*	**	**	**	*	10
Hommel 2016 [22]	*	/	/	**	**	**	*	8
Kamaruzzaman 2010 [38]	*	*	*	*	*	**	*	8
Kartheek 2011 [51]	/	/	/	*	/	**	*	4
Klanbut 2018 [28]	*	/	/	*	**	**	*	7
Kleipool 2019 [29]	*	*	/	*	**	**	*	8
Lampela 2013 [67]	*	*	/	*	*	**	/	6
Liepelt Scarfone 2015 [39]	*	*	/	*	**	**	*	8
Liu 2016 [40]	*	*	/	/	**	**	*	7
Luukinen 1999 [68]	*	*	*	**	**	**	*	10
Luukkonen 2017 [69]	*	*	*	*	**	**	*	9
Mader 1987 [70]	*	*	*	/	*	**	*	7
Masaki 1998 [71]	*	*	*	*	/	**	*	7
Masuo 1996 [41]	/	/	/	/	/	**	*	3
Mendez 2018 [52]	*	*	/	**	**	**	*	9
Merola 2016 [30]	/	*	/	*	*	**	*	6
Nguyen 2017 [53]	*	*	/	*	*	**	*	7
O’Connell 2015 [72]	*	*	/	/	**	**	*	7
Oishi 2016 [42]	/	/	/	*	**	**	*	6
Ong 2017 [73]	*	*	/	/	*	**	*	6
Perez Orcero 2016 [43]	/	*	/	/	**	**	*	6
Putnam 2018 [54]	*	*	*	**	*	**	*	9
Rockwood 2012 [55]	*	*	/	**	*	**	*	8
Romagnolo 2019 [31]	*	/	*	**	**	**	*	9
Shin 2004 [74]	*	*	*	*	**	**	*	9
Sonnesyn 2009 [32]	/	*	/	*	*	**	/	5
Valbusa 2012 [24]	*	*	/	*	**	**	*	8
Vanhanen 1996 [75]	*	*	/	*	*	**	*	7
Van Hateren 2012 [44]	*	*	/	*	**	**	*	8
Velilla Zancada 2017 [76]	*	*	/	*	**	**	*	8
Veronese 2014 [56]	*	*	*	**	**	**	*	10
Viramo 1999 [77]	*	*	/	*	*	**	/	6
Walczak 1991 [45]	/	/	/	*	/	**	*	4
Wecht 2016 [80]	/	*	/	**	**	**	*	8
Wolters 2016 [57]	*	*	*	*	**	**	*	9
Wu 2009 [78]	*	*	/	*	**	**	*	8
Yap 2008 [79]	*	*	*	**	**	**	*	10
Zhu 2016 [46]	/	*	*	*	*	**	*	7

### Reported prevalences

**Fig. 2:**
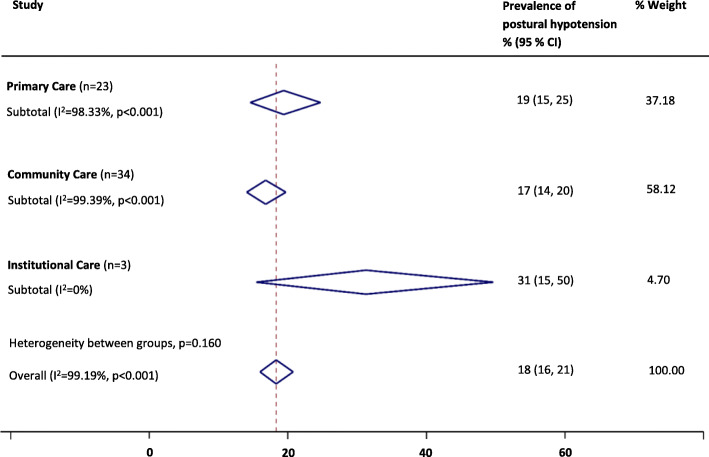
Summary of the prevalence of postural hypotension according to the consensus definition across care settings

Overall, PH prevalence using the consensus definition was 18% (95% confidence interval, 16–21%, I^2^ = 99%). Pooled prevalences of PH were 17% (14–20%; I^2^ = 99%) for 34 community cohorts, 19% (15–25%; I^2^ = 98%) for 23 primary care cohorts and 31% (15–50%; I^2^ = 0%) for three nursing/residential care home cohorts (*P* = 0.16 for between group differences, see Fig. 2). When low quality studies were omitted from analyses, pooled prevalences of PH were 18% (15–23%; I^2^ = 99%) for 20 community cohorts, 22% (18–26%; I^2^ = 93%) for 10 primary care cohorts and 20% (17–22%; I^2^ = 0%) for two nursing/residential care home cohorts (*P* = 0.38 for between group differences).

**Fig. 3:**
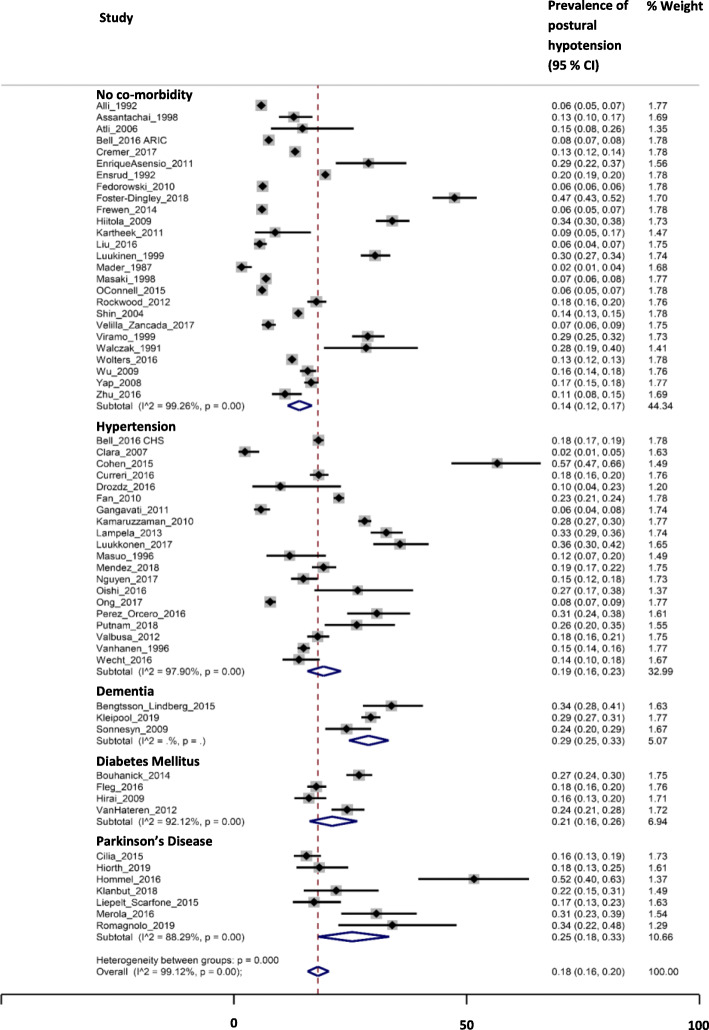
Prevalence of postural hypotension according to the consensus definition across disease subgroups. ‘Control’ group represents those individuals with no co-morbidity

For disease subgroups, pooled prevalences of PH were 19% (16–23%; I^2^ = 98%) in hypertension (20 cohorts), 21% (16–26%; I^2^ = 92%) in diabetes (four cohorts), 25% (18–33%; I^2^ = 88%) in Parkinson’s disease (seven cohorts) and 29% (25–33%; I^2^ = 0%) in dementia (three cohorts), compared with 14% (12–17%; I^2^ = 99%) for those without these conditions (26 cohorts; *P* < 0.01 for between group differences; Fig. 3.). When low quality studies were omitted from analyses, pooled prevalences of PH were 21% (17–26%; I^2^ = 98%) in hypertension (10 cohorts), 21% (16–26%; I^2^ = 92%) in diabetes (four cohorts), 29% (16–44%; I^2^ = 91%) in Parkinson’s disease (four cohorts) and 29% (27–31%; I^2^ = 0%) in dementia (one cohort), compared with 17% (13–21%; I^2^ = 99%) for those without these conditions (13 cohorts; *P* < 0.01 for between group differences).

**Fig. 4:**
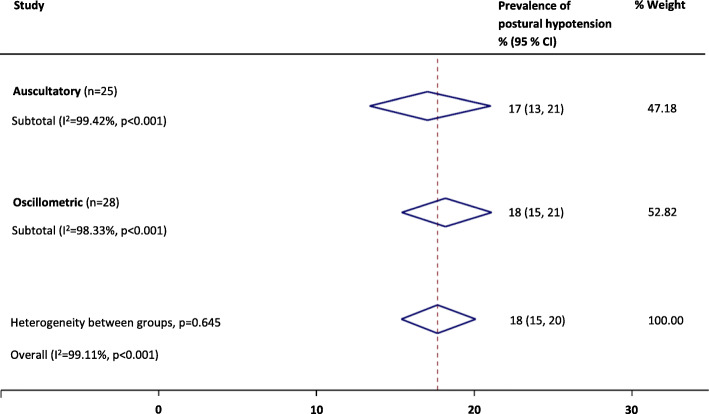
Summary of the prevalence of postural hypotension according to the consensus definition across different measurement methods (auscultatory vs. oscillometric techniques)

**Fig. 5:**
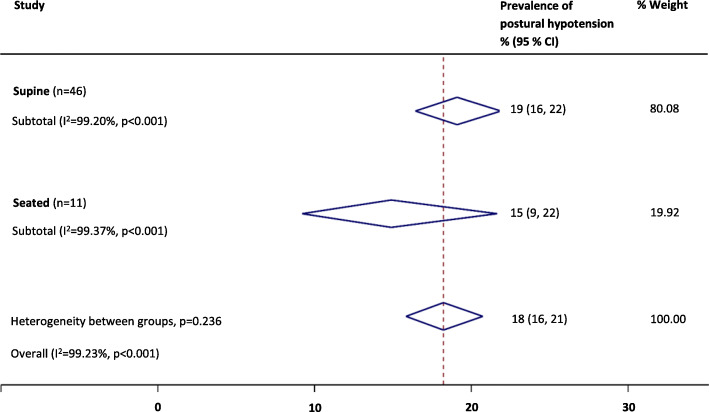
Summary of the prevalence of postural hypotension according to the consensus definition across different resting positions (supine vs. seated techniques)

**Fig. 6:**
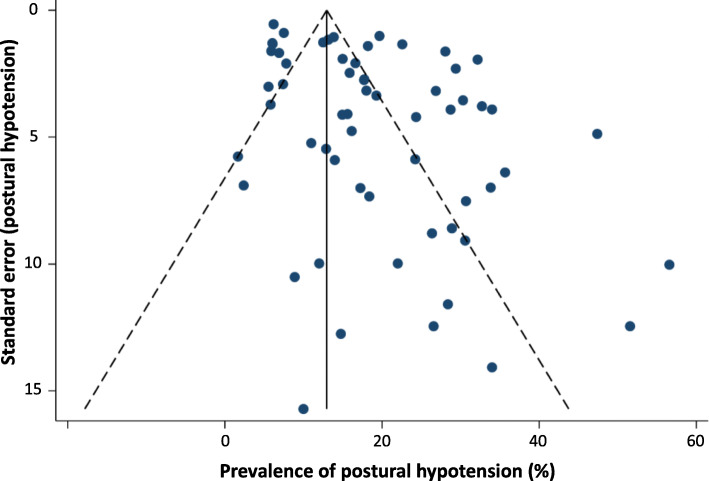
Funnel plot for prevalence of postural hypotension (defined as a drop in systolic blood pressure of ≥20 mmHg or diastolic blood pressure of ≥10 mmHg within three minutes of rising to a standing position). Egger test (*P* < 0.01)

Where the consensus definition of PH was reported at study level, prevalence estimates were higher (23%; 19–27%) than those definitions of PH that were not reported as the consensus definition, but fell within the scope of the definition at study level (16%; 14–19%; *P* = 0.01); this finding persisted on exclusion of low quality studies (*P* = 0.04). Sensitivity analyses revealed that the overall PH prevalence was not significantly affected by the type of BP measurement device [auscultatory, 17% (13–21%) or oscillometric, 18% (15–21%); *P* = 0.65, see Fig. 4.], or when measured from a seated (15%; 9–22%) rather than supine (19%; 16–22%) resting position (*P* = 0.24, see Fig. 5.). When low quality studies were omitted, there remained no difference in PH prevalence between seated (22%; 13–34%) and supine (20; 16–24%) BP measurement methods (*P* = 0.67). Heterogeneity remained high across all subgroups (e.g. setting, disease, PH definition and measurement method) and was not explained by the sensitivity analyses according to study quality. Egger tests (*P* < 0.01) and visual inspection of funnel plots suggested possible publication bias against low prevalence small studies (Fig. 6).

**Fig. 7:**
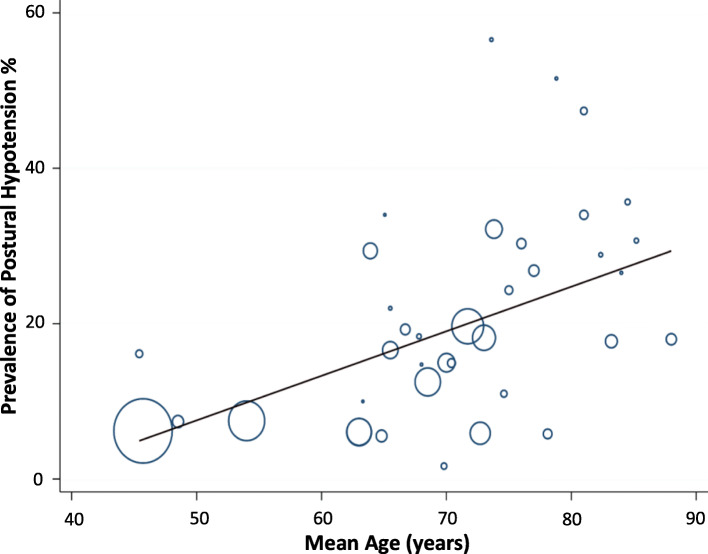
Bubble plot of study level association between mean age and prevalence of postural hypotension according to the consensus definition (systolic ≥20 mmHg or diastolic ≥10 mmHg within three minutes of standing). Circles represent estimates from each study, sized according to precision of estimate

Univariable meta-regression showed three study level factors to be associated with mean prevalence of PH: age (*P* < 0.01), history of falls and disease status (all *P* < 0.05, see Table 4). For multivariable analysis, age (Fig. 7.) and presence of diabetes remained as predictors of PH (*P* < 0.01, *P* = 0.13, respectively; R^2^ = 36%).

**Table 4:** Univariable regression-analyses

Variable	***P***-value	95% CI	Coefficient	Studies reporting variable (n)
Age	*P* = 0.001	0.002 to 0.009	0.0057	38
Gender	*P* = 0.086	−0.000 to 0.004	0.0016	55
Diabetes	*P* = 0.434	−0.000 to 0.002	0.0005	42
Hypertension	*P* = 0.153	−0.000 to 0.002	0.0009	49
BMI	*P* = 0.863	−0.032 to 0.038	0.0028	13
Coronary heart disease	*P* = 0.161	−0.003 to 0.014	0.0057	8
Stroke	*P* = 0.336	−0.005 to 0.014	0.0045	21
Parkinson’s	*P* = 0.993	−0.001 to 0.001	0.0000	19
Falls	*P* = 0.025	0.001 to 0.014	0.0073	12
Setting	*P* = 0.437	−0.187 to 0.043	0.0121	60
Disease status	*P* = 0.033	0.002 to 0.381	0.0198	60
Blood pressure position	*P* = 0.470	−0.043 to 0.091	0.0243	58
Blood pressure device	*P* = 0.869	−0.059 to 0.069	0.0053	53
Seated systolic blood pressure	*P* = 0.797	−0.008 to 0.009	0.0009	7
Seated diastolic blood pressure	*P* = 0.530	−0.039 to 0.227	0.0008	7
Supine systolic blood pressure	*P* = 0.129	−0.003 to 0.165	0.0067	6
Supine diastolic blood pressure	*P* = 0.265	−0.189 to 0.052	0.0165	6

*95% CI* 95% confidence interval

## Discussiones

### Summary

This is, to our knowledge, the first systematic review and meta-analysis to present estimates of PH prevalence in populations regularly encountered in primary care, including general practices and related outpatient clinics, care or nursing homes and community settings. Our findings confirm that PH, when tested for, is a common finding across care settings and disease subgroups, with the highest prevalences observed in people residing in care/nursing homes (and primary care, when low quality studies were omitted from analyses), and in those with dementia; age itself appears to be the key predictor of prevalence. The definition of PH used can impact prevalence estimates and therefore must be considered carefully in clinical practice. The type of BP measurement device and resting position does not appear to systematically impact PH prevalence estimates.

### Strengths and limitations

This study provides insight into PH prevalences across a variety of care settings and disease cohorts. Our search terms were intentionally broad, thus it is unlikely that substantial numbers of relevant publications were overlooked. Data extraction was limited to English language papers and published records, although non English language and Grey literature data generally have been shown to make limited impact on review findings where a substantial body of published evidence exists [81]. We found some evidence for publication bias against low prevalence small studies; overall, there was considerable heterogeneity of PH prevalence estimates across different care settings, disease cohorts, PH definitions and BP measurement methods that was not accounted for in our sensitivity analyses. The utility of the NOS for assessing study quality has previously shown poor agreement between reviewers, with calls for more specific guidance in its use [82]. We adapted the generic guidance to give context specific to this PH review (Appendix 2), however, we did not find any substantial impact on heterogeneity in subgroup analyses according to quality assessment of studies. High residual levels of heterogeneity limit our ability to draw firm conclusions from the data. PH prevalence varied widely across studies (2.0–56.8%) and residual heterogeneity probably reflects cumulative effects of non-systematic variations in population size and health status, limitations in classifying cohorts by condition at study level, and the discrepancy in PH definitions and measurement methods employed across studies.

Our univariable meta-regression showed that the presence of disease was associated with increasing PH prevalence, according to condition. This association did not persist when multivariable regression was undertaken, but there was co-linearity of disease status with diabetes, which was included a priori in the multivariable model. Increasing BP per se, a known risk factor for orthostatic hypotension [83], was not associated with increasing PH incidence; however, data for baseline BP were, surprisingly, only reported in 13 studies, limiting our ability to explore this association. The relationship of PH with hypertension is complex; PH is associated with both uncontrolled hypertension and the number of antihypertensive drugs used in managing high BP [25, 38, 84, 85], but effective treatment of high BP in elderly persons is associated with reduced PH prevalence [41, 86]. Consequently, a non-linear or ‘U’ shaped relationship of prevalence to absolute BP might be expected, with interaction in analyses between a diagnosis of hypertension (indicating treatment) and absolute BP values. Exploration of such a relationship was not possible in the current analyses.

Current guidelines for postural hypotension management recommend clinicians undertake a comprehensive medication review if systolic BP falls by 20 mmHg on standing [6]. The de-escalation of antihypertensive medication is a common treatment method and may increase the probability of recovery from postural hypotension with no increased risk of adverse cardiovascular events [87], however, further work in this area is required.

### Comparison with existing literature

This review builds on existing reviews that have summarised prevalence of PH in specific cohorts, such as those with diabetes or Parkinson’s disease and individuals over 60 years of age [13, 88–91]. Here, we report that PH affects 18% of individuals across care settings and disease cohorts. Our data show that PH incidence rises from community care settings to those attending primary care and residing in institutions. These findings reflect the likelihood that multimorbidity, and the subsequent risk of PH, is more common in care/nursing home settings than general practices or in the community [92]. We also found that individuals with chronic disease have increased prevalence estimates of PH compared to groups without such diseases present. This may be due to a number of factors, including medication (e.g. diuretics, antihypertensives), development of peripheral and/or autonomic neuropathy (associated with diabetes mellitus and dementia) or physical deconditioning (due to age-related changes or continued bed rest) [4].

There appears to have been an exponential rise in interest in PH, with ~ 70% of the studies reported in this review published in the last decade and ~ 50% in the last 5 years. This may reflect interest in rising longevity, multimorbidity and rates of diabetes (risk markers for PH) [93, 94]. Recent reporting of improved cardiovascular outcomes with intensive lowering of BP is also relevant [95, 96], given the risks of adverse events such as PH and falls, associated with lower BP targets [97].

Our findings are consistent with studies that have reported high prevalences of PH in individuals with diabetes (type 1, 19% and type 2, 20%) [88], and in the aged [98]. Prevalences approaching 50% have been reported in Parkinson’s disease with low prevalence of orthostatic symptoms, making the case for routine postural BP testing when reviewing all sufferers [99].

On subgroup analyses, we found no significant difference in PH prevalence when measuring BP in the sitting position rather than supine, prior to standing, and this finding remained on exclusion of low quality studies. This approach may therefore be justified as an alternative to the gold-standard supine-to-stand approach, if undertaken with rigid methodology. Shaw et al. have previously suggested that the sit-to-stand method is a good alternative for busy clinicians when the supine-to-standing method cannot be achieved; they proposed reducing diagnostic thresholds for PH to a systolic drop ≥15 mmHg or a diastolic drop ≥7 mmHg to maximise the sensitivity and specificity of the test and to reflect the reduced orthostatic stress of moving from sitting to standing, compared with lying supine [100]. We found no evidence to support a change in diagnostic threshold in this review, but suggest future studies should directly compare supine versus seated followed by standing PH measurement methods. We also found that adopting auscultatory or oscillometric methods of measuring BP did not impact prevalence estimates. Further work is required across larger cohorts to determine the most appropriate diagnostic criteria for PH in primary care if the pragmatic sit-to-stand method is to be adopted.

When the definition of PH did not fulfil the consensus description, but fell within its parameters, we found that prevalence was underestimated irrespective of study quality. This highlights the importance of adopting the consensus definition to minimise under-detection of PH whenever possible [1].

### Implications for research and/or practice

Our univariable regression analyses confirmed that an increasing PH prevalence is strongly associated with increasing age, with age-related chronic diseases and with previous falls. Multivariable analyses revealed that increasing age and presence of diabetes were particularly associated with increased PH prevalence; such individuals may benefit from routine checking for postural hypotension. The population is aging [101], and people are living for longer periods in older age with levels of dependency, or in care settings [102]. European hypertension guidelines, recommend checking for PH in older people, and this will include greater numbers, with attendant workload pressures, over time [7, 16]. By describing the commonly encountered disease states and care settings associated with higher than background prevalences of PH, we provide evidence to encourage improved recognition of this condition through targeted testing. Ideally, BP should ideally be measured from supine to standing using auscultatory methods and our results support the use of the consensus definition [1, 80]. Pragmatically, however, the sit-to-stand method may also be employed as an alternative to the gold standard if the methods are rigorous [100]. However, further work comparing supine versus seated followed by standing measurement methods should be undertaken to clarify the most approach resting positions and thresholds for accurate PH diagnosis.

## Conclusion

Overall, these findings demonstrate the substantial prevalence of PH across a range of populations and care settings relevant to primary care. Our prevalence findings suggest that checking for the presence of PH should be routinely considered when treating chronic conditions, such as diabetes, particularly in older persons. Failure to follow the consensus definition of PH appears to underestimate prevalence, therefore we advocate adoption of the consensus as a standard whenever checking for PH. Further work is needed to confirm the diagnostic thresholds for postural hypotension when BP is measured in the seated rather than supine position.

## Data Availability

The datasets used and/or analysed during the current study are available from the corresponding authors on reasonable request.

## Appendix 1

### Search strategy (Medline and Embase)

- 1 postural hypotension.mp. [mp = ti, ab, hw, tn, ot, dm, mf, dv, kw, fx, nm, kf, px, rx, ui, sy]
- 2 orthostatic hypotension.mp. [mp = ti, ab, hw, tn, ot, dm, mf, dv, kw, fx, nm, kf, px, rx, ui, sy]
- 3 prevalence.mp. [mp = ti, ab, hw, tn, ot, dm, mf, dv, kw, fx, nm, kf, px, rx, ui, sy]
- 1 or 2
- 3 and 4
- Limit 5 to human

## Appendix 2

### Newcastle-Ottawa Scale adapted for cross-sectional studies for review of postural hypotension prevalence

**Selection:** (Maximum 5 stars (5 points))

Covidence key: 0 stars = High ROB

1 or 2 stars = Low ROB

Cannot tell = Unclear ROB

1. Representativeness of the sample:
  - Truly representative of the average in the target population. * (all subjects or random sampling)
  - Somewhat representative of the average in the target population. * (non-random sampling)
  - Selected group of users.
  - No description of the sampling strategy.
2. Sample size:
  - Justified and satisfactory (a subjective judgement). *
  - Not justified (important mainly if low sample size e.g. < 100).
3. Non-participants:
  - Comparability between respondents and non-respondents characteristics is established, and the response rate is satisfactory. *
  - The response rate is unsatisfactory, or the comparability between respondents and non-respondents is unsatisfactory.
  - No description of the response rate or the characteristics of the responders and the non-responders.
4. Ascertainment of the exposure (i.e. measurement of sitting/lying and standing blood pressure):
  - Validated measurement tool. **
  - Non-validated measurement tool, but the tool is available or described.*
  - No description of the measurement tool.

**Comparability:** (Maximum 2 points)

1. The subjects in different outcome groups are comparable, based on the study design or analysis. Confounding factors are controlled. In this context it is controlling for co-variates.

Reported prevalence may be reported as unadjusted (usual) with logistic regressions or adjusted. Can indicate which in extraction. Go for unadjusted or adjusted?

- The study controls for the most important factor (systolic BP or Age probably both important). *
- The study control for any additional factor (candidates include BMI, BP, age, gender use of antihypertensive medication). *

**Outcome:** (Maximum 3 stars)

The outcome for this review is the prevalence of postural hypotension:

1. Assessment of the outcome:
  - Independent blind assessment. **
  - Record linkage. **
  - Self report. *
  - No description.

These descriptors are unhelpful so suggest re-classify as:

- presented as n/N or proportion for each relevant group in results. **
- Self report i.e. in some way reported by unblinded investigators. *
- No description

2) Statistical test:

In this review – the calculation of proportion(s) with postural hypotension, so should be clearly derived from n/N without unexpected or unexplained omissions from numerator or denominator.

- The statistical test used to analyze the data is clearly described and appropriate, and the measurement of the association is presented, including confidence intervals and the probability level (*p* value). *
- The statistical test is not appropriate, not described or incomplete.

## Abbreviations

PH: Postural hypotension
BP: Blood pressure
NOS: Newcastle-Ottawa Scale
